# Clinical Use of Canine Thawed Refrigerated Plasma: A Historical Case Series

**DOI:** 10.3390/ani13122040

**Published:** 2023-06-20

**Authors:** Weiqin Chee, Claire R. Sharp, Corrin J. Boyd

**Affiliations:** 1 School of Veterinary Medicine, Murdoch University, Murdoch, WA 6150, Australia; 2 Western Australian Veterinary Emergency and Specialty, Success, WA 6164, Australia

**Keywords:** anticoagulant rodenticide, allergic reaction, blood bank, coagulation, haemorrhage, transfusion, rotational thromboelastometry, transfusion reaction

## Abstract

**Simple Summary:**

Thawed plasma (TP) refers to defrosted fresh frozen plasma stored refrigerated. TP is used in human medicine for the rapid provision of clotting factors for the treatment of life-threatening bleeding but its use in dogs has been poorly described. The objectives of this historical case series were to describe the reasons for TP transfusion, treatment outcomes, and adverse events associated with canine TP transfusions in a veterinary teaching hospital. We hypothesised that TP would be used most commonly for the treatment of haemorrhage secondary to anticoagulant rodenticide intoxication and trauma. Blood bank plasma transfusion logs were searched to identify dogs that received at least one unit of TP. Briefly, 166 dogs received a total of 262 units of TP. Anticoagulant rodenticide intoxication (37/166, 22.3%) was the most common reason for transfusion, followed by traumatic haemorrhage (23, 13.9%) and spontaneous haemoperitoneum (22, 13.2%). The majority of dogs received one unit of TP (111/166, 67.1%) and packed red blood cells (pRBCs) were commonly simultaneously transfused with TP (65, 39.2%). Severe prolongations of clotting times were reduced following TP transfusions. Allergic reactions were the most common transfusion reaction (19/166, 11.4%). Most dogs survived to discharge (101/166, 60.8%).

**Abstract:**

Thawed plasma (TP) refers to defrosted fresh frozen plasma stored refrigerated. TP is used in human medicine for the rapid provision of coagulation factors and resuscitation of haemorrhagic shock, but its use in dogs is poorly described. The objectives of this historical case series were to describe the reasons for TP transfusion, treatment outcomes, and adverse events associated with canine TP transfusions in a veterinary teaching hospital. We hypothesised that TP would be used most commonly for the treatment of haemorrhage secondary to anticoagulant rodenticide intoxication and trauma. Blood bank plasma transfusion logs were searched to identify dogs that received at least one unit of TP between December 2015 and June 2021. Briefly, 166 dogs received a total of 262 units of TP. Anticoagulant rodenticide intoxication (37/166, 22.3%) was the most common reason for transfusion, followed by traumatic haemorrhage (23, 13.9%) and spontaneous haemoperitoneum (22, 13.2%). The majority of dogs received one unit of TP (111/166, 67.1%) and pRBCs were commonly simultaneously transfused with TP (65, 39.2%). Severe prolongations of prothrombin time and activated partial thromboplastin time were reduced following TP transfusions. Allergic reactions were the most common transfusion reaction (19/166, 11.4%). Most dogs survived to discharge (101/166, 60.8%).

## 1. Introduction

Canine plasma transfusions are indicated for the rapid replenishment of coagulation factors as part of the treatment of active haemorrhage, and the treatment of inherited or acquired coagulopathies causing bleeding or prior to invasive procedures. Plasma is a scarce resource commonly stored as fresh frozen plasma (FFP), at or below −18 °C, to preserve coagulation factors for up to 12 months from time of collection [1]. The time needed to defrost a unit of FFP represents a significant and sometimes unacceptable delay in treatment, taking approximately 15 min in a running water bath [2] and 30 min using a conventional warm water bath [3].

As haemorrhage is the leading cause of death in human trauma patients, and requires rapid coagulation factor replenishment, certain human hospitals stock plasma products at refrigerator temperatures (4 °C). These include liquid plasma (LP), which has never been frozen, and thawed plasma (TP), defined as defrosted FFP stored refrigerated to facilitate more rapid transfusion, particularly in those requiring massive transfusions [4,5]. Despite current guidelines from the American Association of Blood Banks indicating that TP is only to be stored for up to 5 days, due to the degradation of labile coagulation factors [6], human ex vivo studies have indicated that Factor(F)V and FVIII activity remain within the reference range for 10–14 days [7,8]. Previous ex vivo veterinary studies on canine TP have showed similar trends with adequate coagulation factor activity for up to 35 days post-thaw [3,9,10]. Using a 50% cut off for coagulation factor activity, a recent ex vivo study examining the effects of prolonged refrigerated storage indicated that canine TP was viable for the correction of coagulopathies for up 28 days [10]. Apart from these ex vivo studies, the clinical use of canine TP has not been thoroughly documented, with only one small-scale clinical study reported in abstract form [11].

The objectives of this historical case series were to describe the reasons for plasma transfusion, treatment outcomes, and adverse events associated with canine TP transfusions in a veterinary teaching hospital. Our hypothesis was that TP would be used most commonly for the treatment of haemorrhage secondary to anticoagulant rodenticide intoxication and trauma.

## 2. Materials and Methods

Plasma transfusion logs of the blood bank at The Animal Hospital at Murdoch University (TAHMU) from December 2015 to June 2021 were used to identify dogs that received at least one unit of TP. The logs were also used to determine whether any TP was not utilised and discarded after reaching its expiry date. The electronic and physical medical records of each included dog were subsequently reviewed by a single author (W.C.) to obtain relevant data. Exclusion criteria included incomplete medical records or inconsistencies between the patient medical record and blood bank record with regard to which blood products were administered. Only the first hospitalisation was considered for dogs that received TP transfusions on separate hospitalisations during the study period.

The study period commenced in December 2015 as this was the first time that the blood bank at TAHMU stocked TP. Blood collection and processing to produce plasma was as described in a previous study conducted at the authors’ institution [10]. According to a standard operating procedure (SOP) in our hospital, TP was prepared from FFP by thawing in a 37 °C warm water bath until it was completely liquid, and after which it was subsequently stored at 4 °C in a dedicated blood bank refrigerator. The target stock level at any time was two units of TP: either two units of Dog Erythrocyte Antigen (DEA) 1 negative, or one unit of DEA-1-negative and one unit of DEA-1-positive TP. From December 2015 to the end of January 2020, the SOP dictated a 14-day expiry date (from the date of thawing) based on a previous study [3]. The SOP was updated such that from February 2020 onwards, the expiry date was extended to 28 days based on new data demonstrating acceptable coagulation factor activity or concentration (of FV, VII, VIII, IX, X, vWF:Ag, and fibrinogen) for at least 28 days [10]. Between July 2017 and December 2018, 50% of the blood entering the blood bank was leukoreduced (LR) prior to centrifugation as part of a prospective clinical trial that focused on the effects of leukoreduction on the packed red blood cell component [12]. At all other times, all of the plasma was non-LR. For the purposes of this study, LR TP units were not considered separately from the non-LR TP.

Data collected regarding the recipients of TP transfusions included signalment (age, sex, breed), body weight on admission, reason(s) for transfusion, number of units of TP administered, details of other blood products administered, blood typing results, and pre- and post-transfusion coagulation testing. Additionally, the occurrence and nature of transfusion reactions, treatments provided for transfusion reactions, outcome, and duration of hospitalisation were recorded. Data on transfusion administration rates (e.g., mL/h or mL/kg/h) were not included due to wide variability, depending on patient clinical status, as well as missing data. Adverse reactions were defined according to the Transfusion Reaction Small Animal Consensus Statement (TRACS) [13]. Duration of hospitalisation was determined by the time periods for which the dog had been billed for hospitalisation, in 12 h increments. Outcome was denoted as survived to discharge, died naturally in hospital, or euthanised.

Blood typing of recipients and donors is routinely performed in our hospital using an immunochromatographic strip specific for the presence or absence of the DEA 1 antigen (LabTest BT DEA 1, Alvedia, Limonest, France). Coagulation tests commonly utilised in our hospital include measurement of prothrombin (PT) and activated partial thromboplastin time (aPTT), platelet function testing using PFA100^®^ (Platelet Function Analyser (PFA)—100, Siemens Healthcare, Marburg, Germany), and viscoelastic testing using ROTEM^®^ (ROTEM Delta, Werfen, Bedford, MA, USA) with INTEM and EXTEM reagents. During the study period, the measurement of PT/aPTT was available 24 h/day, while access to PFA100 and ROTEM was limited to daytime hours. During the study period, two different in-house coagulation analysers were used to measure PT and aPTT, each with separate reference intervals. The first coagulation analyser (Abaxis VetScan VSPro, Zoetis, Union City, CA, USA) was used from December 2015 to 12 July 2016. From 13 July 2016 onwards, the second coagulation analyser (SCA2000 Veterinary Coagulation Analyser, Synbiotics, San Diego, CA, USA) was used. Coagulation testing performed by referring veterinarians was also included, with results interpreted based on their reference intervals. The degree of prolongation of PT and aPTT relative to the machine-specific reference intervals was used to categorise each test result as either within the reference interval, mildly prolonged (up to 25% prolonged from the upper limit of the reference interval), moderately prolonged (25% prolonged up to the upper detection limit), or markedly prolonged (greater than the upper limit of detection). The PT reference interval for the first coagulation analyser (Abaxis VetScan VSPro, Zoetis, Union City, CA, USA) in the authors’ institution was 11–17 s (s). The PT reference interval for the second coagulation analyser (SCA2000 Veterinary Coagulation Analyser, Synbiotics, San Diego, CA, USA) was 14–19 s. Both analysers had an upper limit of detection for PT of 35 s. The aPTT reference interval for the first coagulation analyser (Abaxis VetScan VSPro, Zoetis, Union City, CA, USA) was 72–102 s. For the second coagulation analyser (SCA2000 Veterinary Coagulation Analyser, Synbiotics, San Diego, CA, USA), the reference interval for aPTT was 75–105 s. Both analysers had an upper limit of detection for aPTT of 200 s.

An electronic data capture system designed for medical research was used to collate the data [14] and perform descriptive statistical analysis. Continuous variables are presented as median (minimum–maximum). Categorical data are reported as number (percentage); where data were not available for the entire sample, the denominator is provided.

## 3. Results

### 3.1. Dog Population

The search strategy identified 172 dogs that had received at least one unit of TP. Four dogs were excluded due to missing PT/aPTT data with a further two being excluded having presented twice during the study period, leaving 166 dogs for analysis. The median age was 6.03 years (0.16–16 years). The majority of dogs were male neutered (70, 42.2%), followed by female spayed (60, 36.1%), male intact (18, 10.8%), and female intact (18, 10.8%). Forty-six dog breeds were included, with mixed-breed dogs being the most common (57, 34.3%). The most commonly represented pure breeds, with more than five dogs each, included the Staffordshire bull terrier (10, 6.0%), Labrador retriever (8, 4.8%), blue heeler/cattle dog (6, 3.6%), border collie (6, 3.6%), dachshund (6, 3.6%), and German shepherd (5, 3.0%). The median body weight was 20.65 kg (1.17–75 kg).

### 3.2. Reason for Transfusion

The most common reason for TP transfusion was for the treatment of acquired coagulopathy secondary to anticoagulant rodenticide intoxication (37, 22.3%). The reasons for plasma transfusion and associated patient outcomes are displayed in Table 1. The vast majority of cases received TP transfusion for the treatment of haemorrhage and/or coagulopathy. Two dogs received TP for oncotic support, as part of fluid resuscitation for septic shock. Given that these dogs both had marked hypoalbuminemia (10 and 12 g/L (reference interval 24–38 g/L)), they received TP in addition to crystalloid fluids and vasopressor agents in an attempt to restore normotension.

### 3.3. Number of TP Units and Other Blood Products Transfused

In total, 262 units of TP were transfused during the study period. During the same period, 59 units of TP expired and were discarded, of which 35 units were within the period where there was a 14-day expiry date. The majority of dogs received a single unit of TP (111/166, 67.1%), while 34 dogs received two units (20.5%). The remaining 21 dogs received three or more units, with the maximum being nine units transfused to a single dog. The dog receiving nine units of TP had DIC and multiple organ dysfunction syndrome secondary to heat stroke, and died in hospital despite treatment. One hundred and twelve dogs (67.4%) received other blood products in addition to TP. The most common concomitant blood product used was packed red blood cells (pRBCs) (65, 39.2%). The other combinations of blood products used are shown in Table 2.

### 3.4. Blood Typing

The blood typing results for recipients prior to transfusion were recorded in 131 (78.9%) dogs, with 74/131 (56.5%) being DEA 1 negative. For dogs that did not have a type recorded, 33/35 (94.3%) received DEA-1-negative TP units, 1/35 (2.9%) received a single DEA-1-positive TP unit, and 1/35 (2.9%) received a combination of DEA-1-negative and -positive TP.

### 3.5. Coagulation Testing

Pre-transfusion coagulation testing was performed in 134 (80.7%) dogs. Some dogs had more than one type of pre-transfusion coagulation test performed. Out of the pre-transfusion coagulation testing, PT/aPTT tests were performed the most commonly (85/134, 63.4%), followed by ROTEM (60, 44.8%), PFA-100 (5, 3.7%), and activated clotting time (1, 0.7%). Prior to transfusion, PT was normal in 30/85 (35.3%) of dogs, mildly prolonged in 16 (18.8%), moderately prolonged in 4 (4.7%), and severely prolonged in 35 (41.2%). The aPTT was normal in 7/85 (8.2%), mildly prolonged in 18 (21.2%), moderately prolonged in 14 (16.5%), and severely prolonged in 46 (54.1%).

Fewer dogs had post-transfusion coagulation testing (77/166, 46.4%). The majority of these dogs had normal (24/39, 61.5%) PT, while 10/39 (25.6%) had mildly prolonged, 2 (5.1%) had moderately prolonged, and 3 (7.7%) had severely prolonged PT. Post-transfusion aPTT was normal in 2/39 (5.1%), mildly prolonged in 10 (25.6%), moderately prolonged in 18 (46.2%), and severely prolonged in the remainder (9, 23.1%). Post-transfusion ROTEM was performed in 41 dogs. The summary statistics for the pre- and post-transfusion ROTEM results are listed in Table 3.

### 3.6. Transfusion Reactions

When classified according to the TRACS guidelines, the overall transfusion reaction rate in our study was 23/166 (13.8%). Of the dogs that received non-type-matched TP, 6/35 (17.1%) dogs had transfusion reactions. Allergic reactions were the most common (18), followed by citrate toxicity (3) and febrile non-haemolytic transfusion reaction (FNHTR) (1) alone. One dog had both an allergic reaction and FNHTR. Of the dogs that received TP only, 5/19 (26.3%) developed allergic reactions while none developed citrate toxicity or FNHTR. The three patients that fulfilled the criteria for definite citrate toxicity each received a massive transfusion, had acute hepatic failure (*n* = 2) or hepatic impairment (*n* = 1), AND a decrease in ionised calcium to ≤0.7 mmol/L, AND consistent clinical signs (tachycardia and/or hypotension). However, each of these patients had pre-existing and variable tachycardia and hypotension during resuscitation from shock, and thus the origin of the cardiovascular instability could not be definitively attributed to citrate toxicity as compared to the underlying cause of shock.

A considerable number of dogs experienced reactions that did not meet the TRACS definitions. Eleven other dogs in our study were hypocalcaemic during massive transfusion but did not meet the TRACS criteria for citrate toxicity [13] due to a lack of evidence of hepatic impairment, or inadequate severity of ionised hypocalcaemia (i.e., not ≤0.9 mmol/L) and inability to distinguish the clinical signs of citrate toxicity from those associated with the underlying disease. Nonetheless, the median nadir-ionised calcium concentration in this group was 0.91 mmol/L (min–max 0.61–1.16, reference interval (1.25–1.5 mmol/L)). Two dogs with signs of acute respiratory distress were suspected to have transfusion-related acute lung injury; however, they did not meet the criteria due to a lack of measurable hypoxia. One dog developed ventricular tachycardia following a TP bolus that was resolved with discontinuation and reoccurred when the bolus was restarted. Ultimately, the TP rate was reduced and lignocaine was administered. Two dogs showed ptyalism or nausea that were not associated with other clinical signs or reactions such as allergic reactions, hypocalcaemia, or fever.

When considering transfusion reactions as defined by TRACS, reactions in dogs receiving TP alone occurred in 5/54 (9.3%) dogs, compared to 18/112 (16.1%) dogs receiving TP in combination with other blood products. When considering all transfusion reactions (not just those that fulfilled TRACS definitions), reactions to TP alone occurred in 9/54 (16.7%) transfusions, while dogs receiving TP in combination with other blood products had a transfusion reaction rate of 34/112 (30.4%).

None of the recipients were premedicated prior to their transfusions, while the dogs that met the TRACS criteria for transfusion reactions received one or more interventions in the form of medical treatment or adjustments to the transfusion rate. Specifically, all of the dogs with allergic reactions received chlorpheniramine with one receiving an anti-inflammatory dose of dexamethasone concurrently. In addition, the transfusion was slowed but not stopped in one dog after an allergic reaction, and another dog had the transfusion slowed and then restarted at a lower rate. Calcium gluconate was administered in all of the dogs that fulfilled the TRACS criteria for citrate toxicity. Additionally, calcium gluconate was administered prophylactically for ionised hypocalcaemia, concurrent with an administration of massive transfusion, to reduce the risk of progression to severe and symptomatic hypocalcaemia. One dog with hemoperitoneum and coagulopathy secondary to anaphylaxis developed fever and ionised hypocalcaemia (iCa 0.61 mmol/L), and received chlorpheniramine, dexamethasone, and calcium gluconate. The other dog that experienced FNHTR received chlorpheniramine and had its transfusion stopped and restarted at a lower rate.

### 3.7. Duration of Hospitalisation and Outcomes

The majority of the dogs required hospitalisation for more than 12 h (148/166, 89.2%). Most dogs survived to discharge (101, 60.8%), 50 (30.1%) were euthanised, and 15 (9.0%) died naturally. Case outcomes categorised by reason for transfusion are listed in Table 1.

## 4. Discussion

Our study reports on the reasons for plasma transfusion, outcomes, and transfusion reactions of 166 dogs who received at least a single unit of TP over the period of December 2015 to June 2021, and represents the largest study on the clinical use of canine TP.

Consistent with our hypothesis, the most common reason for the transfusion of TP in our population was for the treatment of anticoagulant-rodenticide-induced coagulopathy, followed by haemorrhage due to trauma. These are similar to indications for FFP in dogs reported in previous studies, where the correction of acquired coagulopathies was the main indication [15,16]. Guidelines for TP use and the lifespan of TP in human medicine varies according to geographical regions and even amongst different healthcare facilities [17]. The most commonly cited indications are for trauma patients requiring massive transfusion, patients with warfarin anticoagulation-related intracranial haemorrhage [18], and bleeding with multiple coagulation factor deficiencies [19]. Ready access to factor concentrates in human medicine is the main reason why the indications are reduced considerably compared to indications for plasma transfusion in veterinary medicine.

It is not surprising that anticoagulant-rodenticide-induced haemorrhage was the most common indication for TP transfusion, given that anticoagulant rodenticides are common canine intoxicants [20,21]. Anticoagulant rodenticides exert their effect by inhibiting vitamin K1-2,3 epoxide reductase needed for vitamin K1 regeneration, and thus prevent the endogenous production of clotting factors II, VII, IX, and X [22]. The rapid replenishment of coagulation factors with plasma-containing products, together with vitamin K1 administration in clinically bleeding patients, is recommended, with a recent retrospective study reporting 36% of non-transfused patients dying or being euthanised prior to discharge, compared to 4% of patients transfused with FFP or FP [23]. These outcome statistics are similar to those in our study, where 8% of dogs with anticoagulant-rodenticide-induced bleeding receiving TP were euthanised or died. Thawed plasma would be expected to be adequate for clotting factor replacement in dogs with anticoagulant-rodenticide-induced bleeding, given that activities of FVII, FIX, and X remain well above 50% activity in TP for at least 42 days [10].

The main advantage of stocking TP in human medicine is to allow for more rapid transfusions in trauma patients with massive transfusion protocols, especially given delays with thawing and the initiation of FFP transfusions [24], as well as evidence advocating for plasma-first resuscitation [25,26]. One prospective study evaluating the pre-hospital administration of TP and pRBCs in air-lifted adult trauma patients identified an improved acid–base status upon arrival to hospital, reduced transfusion volumes during the first 6 and 24 h in hospital, and reduced incidence of substantial bleeding compared to patients that received only crystalloid-based resuscitation protocols. Mortality during the first 6 h was also lower; however, there were no differences in 24 h or 30 day mortality [27]. A more recent randomised trial on the early use of TP in air-lifted human trauma patients at risk of haemorrhagic shock showed a significantly lower 30 day mortality and improved international normalised ratio (INR) [28]. Trauma was the second most common reason for TP transfusion in dogs in our study. Acute traumatic coagulopathy (ATC) has been shown to occur in severely traumatised dogs, with a prevalence ranging from 5.5 to 33.3% [29,30], and the affected dogs required more plasma transfusions [29]. Given the retrospective nature of our study, it was not possible to determine whether the use of TP resulted in a quicker administration of plasma and whether this led to improved outcomes. Indeed, the optimal timing of plasma transfusion for the treatment of haemorrhagic shock in veterinary patients remains unknown. Prospective controlled studies focusing on the effects of stocking canine TP on time to plasma transfusion and potential effects on outcome are needed.

Dogs with spontaneous, non-traumatic haemoperitoneum were also well represented in our study, consistent with the coagulation disturbances known to occur in this population. Previous studies have demonstrated that dogs with spontaneous haemoperitoneum had lower platelet counts, longer mean PT and aPTT, and ongoing fibrinolysis, as measured via tPA-TEG, when compared to healthy age- and breed-matched controls [31]. Similarly, a recent study of dogs with haemoperitoneum and shock identified that approximately two thirds were hypocoagulable based on standard PT/aPTT, fibrinogen concentrations, and ROTEM [32]. While the optimal timing of plasma transfusions for the treatment of haemorrhagic shock in veterinary patients remains unknown, there is conflicting information in human medicine on whether plasma-first resuscitation confers a survival benefit [4].

Nine dogs in our study received TP for the treatment of haemoperitoneum associated with anaphylaxis, of which three dogs were subsequently euthanised. The development of spontaneous hemoperitoneum, marked circulatory shock, and severe prolongations of clotting times has been described in dogs secondary to anaphylaxis following *Hymenoptera* envenomation [33,34,35]. Proposed mechanisms include acute hyperfibrinogenolysis, coagulopathy, and severe vasculitis, associated with the elements of bee venom. While the use of epinephrine and aggressive crystalloid fluid resuscitation forms the mainstay of treatment of the distributive and hypovolemic mechanisms of shock experienced in these patients, the use of blood product transfusions (FFP, pRBCs, and/or autotransfusion) has also been described [33,34]. Further research is needed to determine whether TP is a viable product for the correction of coagulopathy in dogs with spontaneous haemoperitoneum secondary to anaphylaxis.

Two dogs in our case series received TP for the management of congenital coagulopathies: one had confirmed haemophilia A, and the other was suspected to have haemophilia A. The use of TP for haemophilia A has not been reported in dogs, with cryoprecipitate being the preferred product due to the lower volumes required [36,37]. Human TP is not recommended for the treatment of haemophilia A patients with active haemorrhage because FVIII activity declines significantly during refrigerated storage, with up to 45% being lost at the end of 2 weeks [7]. While FVIII activity declines over time in canine TP, FVIII only drops <50% activity by day 32 [10], and thus TP stored for ≤28 days, as in this study, is likely to be a suitable alternative to FFP or cryoprecipitate for the treatment of haemorrhage associated with haemophilia A in dogs.

Two dogs in our study received TP as part of fluid resuscitation for oncotic support, given marked hypoproteinemia and septic shock. The use of plasma for colloid oncotic support is controversial due to the low albumin content of a single unit. A previous study found that the transfusion of a median dose of 15–18 mL/kg of FFP was not associated with a significant increase in post-transfusion serum albumin compared to the pre-transfusion value [15]. Nonetheless, it appeared that TP was not specifically used to increase albumin concentration in the dogs in our study but rather as part of a multimodal approach to the treatment of septic shock. While no guidelines for the management of septic dogs exist, there is weak evidence supporting the use of albumin and crystalloids for resuscitation and intravascular volume replacement in septic humans [38]. Since canine albumin is not available in the authors’ country, the use of plasma may be a reasonable alternative.

Transfusion reaction rates in our study were higher compared to previous studies focusing on plasma transfusions, which reported rates of 1% [15] and 4.1% [16]. One possible reason for this could have been the concurrent use of other blood products such as pRBCs and FWB, which are associated with higher rates of transfusion reactions [16,39]. Underreporting of transfusion reactions is common [40] and might have led to a lower rate in other studies, while the higher frequency of hypocalcaemia in our study might have been from closer monitoring in dogs receiving rapid and large-volume transfusions. Further studies would also be useful to determine whether the rate or volume of TP administration is associated with the development of allergic transfusion reactions. While the TRACS guidelines were used to classify the transfusion reactions observed, some patients developed changes that were thought to be transfusion-related but that did not meet the criteria for existing reactions. These included ionised hypocalcaemia not meeting the criteria for citrate toxicity, and ventricular tachycardia unassociated with an acute haemolytic transfusion reaction (AHTR), citrate toxicity, allergic reaction, or a respiratory reaction. It is likely that a greater awareness of transfusion reactions created by the publication of the TRACS guidelines may result in further research into such reactions, allowing for the refinement of the TRACS definitions over time.

Most of the dogs in our study were blood typed for DEA 1 and received type-matched TP. Only a small number of non-matched patients developed transfusion reactions. This is consistent with the TRACS guidelines, which state that there is currently insufficient evidence regarding the use of type-specific plasma to reduce the risk of transfusion reactions [41].

During the period studied, 59 units of TP were discarded after having met their expiry dates. While this number was likely reduced with the aforementioned evidence-based extension in expiry dates, this might represent a level of wastage unsustainable for most veterinary clinics without a dedicated high-volume blood bank. A retrospective study in a human level 1 trauma centre that initially adopted a TP resuscitation strategy had a lower level of wastage after swapping to a hybrid thawed and LP strategy [42]. However, these findings need to be considered against the limited expiry date of human TP of 5 days compared to LP at 26 days. Based on recent studies demonstrating that some individual coagulation factors in canine TP maintain acceptable activity for more than 42 days (FV, VII, IX, and X) [10], and that TP maintains global haemostatic capacity for at least 35 days [9], this wastage could be reduced further.

Some outcomes in our study were similar to those reported in the literature for dogs treated with FFP (e.g., for anticoagulant-rodenticide-induced haemorrhage); however, survival to discharge was numerically lower in our study for those with spontaneous hemoperitoneum (45.4% vs. 83.3% [43]) and traumatic haemorrhage (50% vs. 87% [44]) compared to the existing literature. The factors contributing to survival outcomes cannot be determined in a retrospective case series, but may be related to the use of TP or other factors such as severity of illness or owners’ choice to euthanise due to poor prognosis, age, or financial considerations [45]. Prospective controlled studies comparing the transfusion requirements and survival outcomes of dogs receiving TP compared to FFP should be performed.

Due to the retrospective nature of this study, the effects of concurrent treatments, and the non-standardised pre- and post-transfusion coagulation testing, the impact of TP on the coagulation test results could not be clearly assessed. A prospective clinical trial with standardised timing of pre- and post-transfusion coagulation testing would be required to directly assess the effects of TP on coagulation. Another limitation is the inclusion of TP units generated after LR, which could have contributed to a lower rate of transfusion reactions that might have otherwise occurred with non-LR plasma. One canine study comparing the effects of autologous transfusion of LR versus non-LR pRBCs demonstrated evidence of systemic inflammation (significant increases in total leukocyte and neutrophil counts, fibrinogen, and C-reactive protein) in the non-LR group [46]. However, the applicability of this to plasma remains unknown. The effects of leukoreduction on coagulation factor activity remain contentious, with one canine study showing that LR plasma led to a significant reduction in FXI activity [47] and another showing no significant difference in coagulation factor activities between LR and non-LR plasma in dogs [48].

## 5. Conclusions

This study reports the reasons for plasma transfusion and outcomes of TP use in dogs. The dogs receiving TP were similar to those in previous studies of canine FFP. We propose that prospective studies of TP be performed to identify the potential benefits associated with reducing the time to transfusion and associated differences in outcome, particularly in rapidly haemorrhaging and haemodynamically unstable patients.

## Figures and Tables

**Table 1:** Reason for transfusion and clinical outcomes in 166 dogs that received thawed plasma. Listed from most common to least common reason.

Reasons for Plasma Transfusion	Number of Dogs (%)	Outcome		
		Discharged	Euthanised	Died
Anticoagulant rodenticide intoxication	37 (22.3%)	34	2	1
Traumatic haemorrhage	23 (13.9%)	11	9	3
Spontaneous haemoperitoneum (due to bleeding abdominal mass)	22 (13.3%)	10	11	1
Acquired coagulopathy—other	19 (11.4%)	8	9	2
Acquired coagulopathy—DIC due to SIRS or sepsis	16 (9.6%)	8	5	3
Surgical haemorrhage or following invasive procedure (FNA, rhinotomy, or biopsy)	16 (9.6%)	11	4	1
Acquired coagulopathy—Anaphylaxis-induced haemoperitoneum	9 (5.4%)	6	3	0
Acquired coagulopathy—liver failure	9 (5.4%)	5	3	1
Acquired coagulopathy—DIC due to heat stroke	5 (3.0%)	2	2	1
Oncotic support	2 (1.1%)	1	1	0
Congenital coagulopathy—suspected or confirmed haemophilia A	2 (1.2%)	2	0	0
Reason unclear from medical records	3 (1.8%)	1	1	1
von Willebrand disease—active bleeding	2 (1.2%)	0	1	1
von Willebrand disease—prophylactic	2 (1.2%)	2	0	0
Excessive bleeding in greyhounds	2 (1.2%)	1	1	0
Bleeding due to immune-mediated thrombocytopenia	1 (0.6%)	0	1	0

Abbreviations used in table: DIC, disseminated intravascular coagulation; FNA, fine needle aspirate; SIRS, systemic inflammatory response syndrome.

**Table 2:** Combinations of blood products administered to 166 dogs that received thawed plasma transfusion.

Combinations of Blood Products Transfused	Number of Dogs (Percentage)
TP only	54 (32.5%)
TP + pRBCs	65 (39.2%)
TP + other products (PRP, CRYO, FP, autotransfusion, human albumin, FWB)	18 (10.8%)
TP + FFP	15 (9.0%)
TP + FFP + pRBC	11 (6.6%)
TP + FP	3 (1.8%)

Abbreviations used in table: TP, thawed plasma; pRBC, packed red blood cells; PRP, platelet-rich plasma; CRYO, cryoprecipitate; FP, frozen plasma; FFP, fresh frozen plasma; FWB, fresh whole blood.

**Table 3:** Pre- and post-transfusion ROTEM results in dogs that received thawed plasma. Data are median (min–max) compared to the institutional reference intervals.

	Pre-Transfusion	Post-Transfusion	Reference Interval
InTEM			
CT (s)	253 (84–2356)	229 (37–852)	113–270
CFT (s)	195 (52–1382)	175 (45–2406)	68–272
MCF (mm)	57 (7–79)	61 (7–278)	42–67
Alpha (0)	46 (10–74)	50 (10–175)	53–76
ML (%)	0 (0–100)	0 (0–5)	-
**ExTEM**			
CT (s)	67.5 (24–2137)	59.5 (24–554)	30–94
CFT (s)	202 (0–1688)	156 (49–2252)	69–249
MCF (mm)	55 (5–85)	61 (20–83)	42–70
Alpha (0)	45 (12–76)	52.5 (5–399)	59–77
ML (%)	1 (0–100)	0 (0–34)	-

Abbreviations: CT, clotting time; CFT, clot formation time; MCF, maximum clot firmness; ML, maximum lysis.

## Data Availability

Data available upon request.
